# Strength of religious faith is associated with altered sense of agency during multisensory conflict

**DOI:** 10.1093/nc/niag041

**Published:** 2026-07-30

**Authors:** Gerardo Salvato, Alara France, Manuela Sellitto, Martina Corgnati, Ugo Volli, Luigi Pizzamiglio, Gabriella Bottini

**Affiliations:** Department of Brain and Behavioral Sciences, University of Pavia, Campus della Salute, Policlinico San Matteo, Viale Golgi 19, 27100 Pavia, Italy; Cognitive Neuropsychology Centre, ASST “Grande Ospedale Metropolitano Niguarda”, Piazza Ospedale Maggiore 3, 20162 Milano, Italy; NeuroMi, Milan Center for Neuroscience, University of Milano-Bicocca, Piazza dell’Ateneo Nuovo, 120126 Milano, Italy; Cognitive Neuropsychology Centre, ASST “Grande Ospedale Metropolitano Niguarda”, Piazza Ospedale Maggiore 3, 20162 Milano, Italy; NeuroMi, Milan Center for Neuroscience, University of Milano-Bicocca, Piazza dell’Ateneo Nuovo, 120126 Milano, Italy; Department of Brain and Behavioral Sciences, University of Pavia, Campus della Salute, Policlinico San Matteo, Viale Golgi 19, 27100 Pavia, Italy; Cognitive Neuropsychology Centre, ASST “Grande Ospedale Metropolitano Niguarda”, Piazza Ospedale Maggiore 3, 20162 Milano, Italy; NeuroMi, Milan Center for Neuroscience, University of Milano-Bicocca, Piazza dell’Ateneo Nuovo, 120126 Milano, Italy; Accademia di Belle Arti di Brera, Via Brera 28, 20121 Milano, Italy; Department of Philosophy and Education Sciences, University of Torino, Via Sant'Ottavio 20, 10124 Torino, Italy; Department of Psychology, “La Sapienza” University of Roma, Via dei Marsi, 78, 00185 Roma, Italy; Department of Brain and Behavioral Sciences, University of Pavia, Campus della Salute, Policlinico San Matteo, Viale Golgi 19, 27100 Pavia, Italy; Cognitive Neuropsychology Centre, ASST “Grande Ospedale Metropolitano Niguarda”, Piazza Ospedale Maggiore 3, 20162 Milano, Italy; NeuroMi, Milan Center for Neuroscience, University of Milano-Bicocca, Piazza dell’Ateneo Nuovo, 120126 Milano, Italy

**Keywords:** religious faith, bodily self-awareness, sense of agency, multisensory illusion

## Abstract

Religious belief systems shape human experience of the self and the world, yet their influence on the neurocognitive mechanisms underlying bodily self-awareness remains poorly understood. The present study investigated whether subjective strength of religious faith modulates components of bodily self-awareness during multisensory conflict. Sixty-two healthy female participants completed the Mirror Box Illusion paradigm, assessing explicit experiences of ownership and agency as well as implicit proprioceptive drift, and self-report measures of religious faith and interoceptive sensibility. While synchronous stimulation reliably enhanced embodiment across participants, subjective strength of religious faith selectively modulated the sense of agency. Specifically, individuals with stronger religious faith showed an illusion of agency even under asynchronous visuomotor stimulation, indicating reduced sensitivity to sensorimotor incongruence. Religious faith level was also negatively associated with interoceptive sensibility; however, interoception did not mediate the relationship between faith and agency. These findings suggest that stronger religious faith is associated with a perceptual weighting strategy that prioritizes external visual cues over motor signals. Overall, our results provide evidence that the level of religious faith may modulate the sensory processing underlying agency.

## Introduction

Across religious traditions, the body is understood as a meaningful and symbolic medium through which moral values, social belonging, and spiritual commitments are expressed. Religion, therefore, provides structured frameworks that shape not only bodily practices but also how individuals experience control, intentionality, and responsibility for their own actions (Furey 2012, Hall and Boyatzis 2016, Brandt 2019). Through normative prescriptions and repeated embodied practices such as fasting, ritual purification, prayer, and modesty rules, religious systems regulate bodily appearance and behavior while encouraging sustained attention to self-monitoring and self-regulation (Böttigheimer and Kamp 2023).

Within many religious traditions, women’s bodies and actions in particular are subject to explicit regulation through norms governing modest dress, veiling, or hair covering, which establish shared expectations of bodily presentation in public space (Droogsma 2007, Gökarıksel and Secor 2010, Fuchs 2012, Loewenthal and Solaim 2016, Merdjanova 2021, Aune et al. 2023, Shufan-Biton 2025). These practices do not merely concern outward appearance but involve continuous negotiation of bodily control, self-discipline, and responsibility for one’s actions. In this sense, religious adherence may shape not only how the body is experienced as one’s own, but also how individuals experience themselves as agents acting through their bodies. By repeatedly directing attention, evaluation, and control toward the body, religious norms may therefore influence how bodily signals are monitored, interpreted, and integrated into bodily self-awareness.

From a neuroscientific perspective, bodily self-awareness arises from the integration of exteroceptive, proprioceptive, and interoceptive signals that sustain a coherent representation of the body in mind (Tsakiris 2010, Blanke 2012, Crucianelli et al. 2013, 2018, Lopez et al. 2008, Ponzo et al. 2018, Preuss and Ehrsson 2019, Ehrsson 2020, Salvato et al. 2020a; Salvato et al. 2025b). This representation involves two core experiences: the sense of ownership, that is the feeling that one’s body belongs to oneself, and the sense of agency, or the feeling of being the author of one’s actions (Tsakiris et al. 2007, Tsakiris 2010, 2011). Experimental paradigms introducing a mismatch between sensory modalities have shown that both ownership and agency can be temporarily altered under such conditions (Botvinick and Cohen 1998, Tsakiris and Haggard 2005, Ehrsson 2007, 2012, Lenggenhager et al. 2007, Slater et al. 2010, Aspell et al. 2012, Medina et al. 2015, Preuss and Ehrsson 2019, Crivelli et al. 2021, Pyasik et al. 2022). These effects can be explained by the relative weighting of different sensory signals during multisensory integration processing (e.g. Sebastiano et al. 2024).

Crucially, individuals differ markedly in their susceptibility to multisensory illusion paradigms. Such variability has been linked to implicit factors related to the processing of internal bodily signals (i.e. interoception) and to more explicit aspects such as higher-level interpretative factors about the body (Apps and Tsakiris 2014, Tsakiris 2017, Thériault et al. 2022). Individuals who are less attuned to internal bodily signals tend to rely more heavily on external sensory cues, particularly vision, and are therefore more likely to experience body ownership illusions (Salvato et al. 2020b, Tsakiris et al. 2011). Conversely, individuals with a stronger or more stable sense of bodily self tend to show greater resistance to multisensory perturbations (Crucianelli et al. 2018). From this perspective, susceptibility to changes in ownership and agency in multisensory illusion paradigms may reflect the extent to which different external and internal sensory information is available or weighted in multisensory processes that shape bodily experience.

Importantly, cultural and social belief systems may systematically influence this weighting by shaping how bodily experience is constructed and regulated. In religious contexts, bodily experience is organized through normative systems governing conduct and moral life, such that religion is lived through the body (Csordas 2002, McCullough and Willoughby 2009, Furey 2012, Höpflinger et al. 2016, Van Cappellen and LePage Drummond 2024). Building on this perspective, religious engagement may influence bodily self-awareness through two partially distinct pathways. On the one hand, sustained engagement with strict rules and practices may shift attentional and perceptual priorities away from internal bodily signals. Consistent with this possibility, individual differences in interoceptive accuracy have been linked to broader ideological orientations, with greater sensitivity to internal bodily states associated with more liberal attitudes and reduced interoceptive attunement associated with more conservative orientations (Ruisch et al. 2022), suggesting that different cultural systems may modulate access to interoceptive information. In line with previous evidence, reduced sensitivity to internal bodily signals is associated with greater reliance on exteroceptive information, particularly vision, thereby increasing susceptibility to multisensory illusions (Tsakiris et al. 2011, Salvato et al. 2025a).

On the other hand, religious belief systems may also promote more stable higher-order representations of the body. Repeated exposure to explicit norms governing bodily conduct, responsibility, and self-discipline may strengthen top-down models of the body and of the self as an intentional agent acting through it, through increased self-monitoring and self-regulatory control processes associated with religiosity (McCullough and Willoughby 2009, Carter et al. 2012). Such stabilization of higher-level bodily representations could constrain multisensory integration processes, thereby enhancing resistance to multisensory perturbations and reducing susceptibility to illusions.

Based on these considerations, the present study examined whether individual differences in religiosity are associated with systematic differences in bodily self-awareness under multisensory illusion paradigm. Religiosity was operationalized through the lens of subjective strength of religious faith, assessed using a standardized self-report measure, independent of specific religious affiliation or practices. To probe the malleability of bodily self-awareness, participants were tested using the Mirror Box Illusion, a well-established paradigm that induces conflict between visual, motor, and proprioceptive signals and allows assessment of explicit experiences of body ownership and agency, as well as limb proprioceptive recalibration. In addition, participants completed a questionnaire assessing interoceptive sensibility, indexing the self-reported tendency to notice and attend to internal bodily sensations.

Within this framework, we tested two alternative hypotheses that map onto these distinct mechanisms. The attenuation hypothesis concerns the relative contribution of internal *versus* external sensory signals and predicts that higher religious faith strength is associated with reduced subjective salience of internal bodily signals and greater reliance on external sensory information, thereby increasing susceptibility to multisensory perturbations of bodily self-awareness. Conversely, the stabilization hypothesis posits that external religious norms strengthen higher-order bodily representations and predicts that greater religious faith strength is associated with more stable bodily self-representations, thereby reducing susceptibility to multisensory illusions.

## Materials and methods

### Participants

An *a priori* power analysis was conducted to determine the required sample size for a repeated measures design (G*Power 3.1; Faul et al. 2007). The analysis assumed a medium effect size (*f* = 0.2), an alpha level of 0.05, and a desired power of 0.95. The results indicated that a minimum of 62 participants would be needed to detect the expected effect with adequate power.

Sixty-two healthy female volunteers (age: *M* = 22.5, SD = 3.2, range = 19–38 years; education: *M* = 14.7, SD = 1.8, range = 12–20 years) participated in the study. All participants were right-handed according to the Edinburgh Handedness Inventory, had normal or corrected-to-normal vision and had no previous history of mental or neurological illness. Participants also reported their religious affiliation: 29 identified as Roman Catholic, 8 as Muslim, and 25 reported no religious affiliation (atheist). The inclusion of non-religious participants allowed us to capture variability across the full spectrum of subjective strength of religious faith.

Participants received course credits for their involvement in the study. Before starting the experiment, they gave their written informed consent. The study was approved by the local Ethics Committee of the Department of Brain and Behavioral Sciences, University of Pavia, and conducted in accordance with established ethical standards for research involving human participants.

### Assessment of subjective adherence to religion

Religiosity is here operationalized as subjective strength of religious faith, as assessed by the Santa Clara Strength of Religious Faith Questionnaire (Plante and Boccaccini 1997). This measure captures the personal importance, salience, and motivational relevance of religious belief, rather than specific doctrinal content, ritual practices, or forms of bodily discipline. As such, the present investigation does not test the effects of particular religious traditions or embodied practices but rather examines how individual differences in the subjective centrality of religious faith relate to multisensory processing and bodily self-awareness. The Santa Clara Strength of Religious Faith Questionnaire (Plante and Boccaccini 1997) is composed by 10 items. Participants are asked to indicate how much they agree or disagree with each statement on a Likert scale (from 1 = strongly disagree to 4 = strongly agree), and it focuses on the personal importance and influence of faith in an individual’s life (e.g. “my religious faith is extremely important to me,” “I pray daily,” “I look to my faith as a source of inspiration”).

In the present sample, religious faith scores showed substantial variability (*M* = 20.23, SD = 9.01), ranging from 10 to 40, indicating that participants spanned a broad portion of the scale. Inspection of the distribution revealed no evidence of ceiling effects and only a modest clustering toward the lower end of the scale. Consistent with this, the distribution was moderately positively skewed (skewness = 0.68), indicating that lower religiosity scores were more frequent, although values spanned a wide range.

### Mirror-Box Illusion paradigm

To assess the malleability of bodily self-awareness, participants finally underwent to a session of Mirror-Box Illusion [MBI; (Ramachandran and Rogers-Ramachandran 2007)] administered as in previous studies (Medina et al. 2015, Crivelli et al. 2021, 2023, Salvato et al. 2025b). The experimental task was designed to induce a mirror-based bodily illusion using a simple and controlled setup. Participants sat in front of a flat wooden board (91.4 cm long × 41.7 cm wide) on which two acrylic mirrors (35.5 cm wide × 30.3 cm high) were mounted in the centre, separated by 15.24 cm. Participants placed both their hands against the mirrors, with each hand positioned behind one mirror. To ensure that participants could not see their real left hand, the left arm was covered with a black cloth. As a result, participants could only see the reflection of their right hand in the mirror, which visually appeared to be where their left hand should be.

Participants were instructed to focus their gaze on the mirror image of their right hand and to perform a simple tapping task with their index fingers. Specifically, they tapped their index fingers against the mirrors while their left hand remained hidden from view. The task included two experimental conditions. In the synchronous condition, which was intended to induce the illusion of ownership over the reflected hand, participants tapped both index fingers at the same time, following a metronome set to 170 beats per minute. In the asynchronous condition, used as a control, participants tapped their fingers at the same tempo but alternated the movements (i.e. when one finger tapped, the other remained still). Each condition lasted 60 seconds.

**Figure 1:**
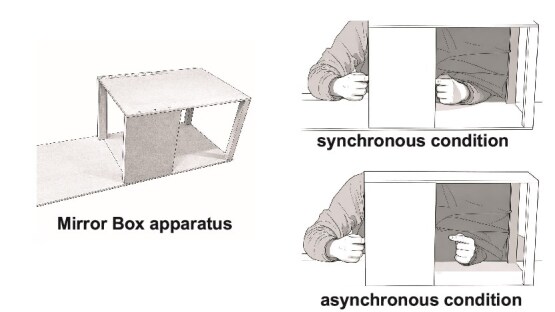
The Mirror Box Illusion. Left panel: schematic illustration of the custom-built mirror box consisting of a flat wooden base with two centrally positioned acrylic mirrors. A horizontal ruler with scrambled numerical labels is mounted on the top surface of the apparatus, in front of the participant, and is used to assess proprioceptive drift. Right panels: participant positioning during the task. The left hand is concealed behind a black cloth beneath one mirror, while the right hand remains visible, creating the visual illusion of the hidden hand. Participants performed index-finger tapping either synchronously (top; both fingers tapping simultaneously to induce the illusion) or asynchronously (bottom; alternating finger movements to serve as a control condition).

After each condition, participants were instructed to stop tapping and to keep both index fingers still at the position reached at the end of the trial, maintaining contact with the mirror. While their left index finger remained hidden from view, participants verbally indicated its perceived position using a horizontal ruler mounted on the top surface of the apparatus, directly in front of them (Fig. 1). The ruler displayed non-sequential (scrambled) numerical labels to prevent participants from relying on visual landmarks or learned spatial mappings when making their judgments. Proprioceptive drift was computed as the difference between the reported position of the hidden left index finger and its actual position, with positive values reflecting a shift toward the location of the visible (reflected) right index finger.

Finally, after each condition, participants completed the Embodiment Questionnaire in a paper-and-pencil format to assess their subjective experience of the illusion (see the appendix for the full questionnaire). The experimenter specified that certain questions referred to the hand in the mirror in the context of ongoing movements, thereby encouraging participants to interpret the reflected hand as representing their unseen hand rather than as a detached mirror image (e.g. in the case of the agency question). The questionnaire comprised eight statements, and participants rated their level of agreement on a Likert scale ranging from +3 (strongly agree) to −3 (strongly disagree). These items were designed to assess five distinct components of embodiment: Ownership, Location, Agency, De-afference, and Affect. Ownership captures the extent to which participants experienced the mirrored hand as part of their own body. Location indexes the degree to which the mirrored hand was perceived as spatially aligned with the participant’s actual hand. Agency describes the extent to which participants felt they could intentionally control the movements of the hand observed in the mirror. De-afference denotes altered sensory qualities in the occluded hand, such as reduced vividness, numbness, or tingling sensations. Finally, Affect concerns the subjective emotional valence of the experience, specifically how pleasant it was.

### Body perception questionnaire

Interoceptive sensibility was assessed using the 22-item Body Perception Questionnaire (BPQ-22; Poli et al. 2021). This self-report measure evaluates subjective sensibility to a broad range of internal bodily sensations, including autonomic reactivity (e.g. heartbeat awareness, respiratory effort, gastrointestinal sensations) and the general monitoring of visceral and bodily states. The questionnaire includes items tapping domains such as cardio-respiratory awareness, digestive sensations, and autonomic arousal (e.g. “I notice changes in my breathing,” “I feel my heart pounding,” “I am aware of stomach or gut tension”). Participants indicated how frequently they experience each bodily sensation on a five-point Likert scale (1 = never to 5 = always). Higher total scores reflect greater self-reported interoceptive sensibility. The BPQ-22 was included to examine whether religious faith strength is associated with differences in interoceptive sensibility and the perception of internal bodily signals. The BPQ-22 was selected as it specifically assesses awareness of physiological and autonomic bodily signals, which are central to the present hypotheses, whereas other measures [e.g. Multidimensional Assessment of Interoceptive Awareness (MAIA)] capture broader and partially distinct dimensions of interoception.

### Statistical analyses plan

To examine the impact of experimental condition and the strength of religious faith on vividness of illusion and proprioceptive drift, two linear mixed-effects models were employed. The first model focused on embodiment questionnaire responses and included fixed effects for *condition* (synchronous, asynchronous), *question type* (affect, agency, deafference, disownership, location, and ownership), *religious faith strength* (Santa Clara Strength of Religious Faith Questionnaire (SCSRFQ) scores), and all interactions, with participants’ ID as a random effect. The second model predicted proprioceptive drift scores with fixed effects for *condition* (synchronous, asynchronous), religious faith strength (SCSRFQ scores), and their interaction, including a random intercept for participants’ ID. *Post hoc* comparisons were adjusted using Holm correction. To explore the relationship between religious faith strength and interoceptive sensibility (BPQ-22 scores) we performed Pearsons’s correlations. Data were analyzed using *JAMOVI* (version 2.6.2) and *R* (version 4.3.1).

## Results

**Figure 2:**
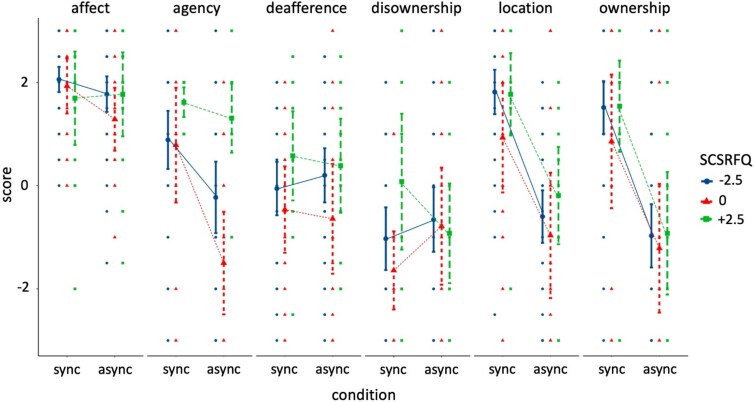
Effects of the strength of religious faith on explicit embodiment ratings in the Mirror Box Illusion. Mean predicted questionnaire ratings for the different embodiment dimensions (ownership, agency, location, affect, deafference, and disownership) as a function of experimental condition and subjective strength of religious faith. A significant three-way interaction between condition, embodiment dimension, and religiosity emerged, driven by a selective modulation of the agency component: at high SCSRFQ levels, participants presented an illusion of agency also in the asynchronous condition. Error bars represent 95% confidence intervals. Different line styles and point symbols denote levels of subjective strength of religious faith (SCSRFQ scores): dotted lines with triangular markers corresponds to the sample mean, dashed lines with square markers to +2.5 SD above the mean, and solid lines with circular markers to −2.5 SD below the mean. Dots represent individual participants’ data points. Sync = synchronous condition; Async = asynchronous condition.

*Embodiment questionnaire.* Results showed an impact of the strength of religious faith on the vividness of illusion questionnaire. There were significant main effects of condition, *F*(1, 682) = 97.93, *P* < .001, question type, *F*(5, 682) = 51.20, *P* < .001, and religious faith strength (SCSRFQ scores), *F*(1, 62) = 4.39, *P* = .040. Participants reported stronger embodiment in the synchronous than in the asynchronous condition (*M* = 0.83, SE = 0.13 versus *M* = −0.14, SE = 0.13), and the effect of condition was significantly modulated by question type, *F*(5, 682) = 22.36, *P* < .001. Holm-corrected post-hoc analyses revealed that synchronous stimulation led to significantly greater ratings than asynchronous stimulation for **ownership**, *t*(682) = 6.16, *P* < .001, (sync: *M* = 1.37, SE = 0.21; async: *M* = −1.02, SE = 0.21), **location**, *t*(682) = 5.62, *P* < .001 (sync: *M* = 1.60, SE = 0.21; async: *M* = −0.60, SE = 0.21), and **agency**, *t*(682) = 5.03, *P* < .001 (sync: *M* = 1.02, SE = 0.21; async: *M* = −0.19, SE = 0.21). Smaller, yet significant effects were also observed for **disownership**, *t*(682) = 2.27, *P* = .024, where ratings decreased in the synchronous condition (sync: *M* = −0.94, SE = 0.21; async: *M* = −0.74, SE = 0.21). No significant differences were found for the **affect** and **deafference** components between conditions (see Fig. 2).

The level of subjective strength in religious faith interacted with question type, *F*(5, 682) = 3.79, *P* = .002, and there was a significant three-way interaction between religiosity, condition, and question type, *F*(5, 682) = 2.55, *P* = .027). To explore the significant three-way interaction between condition, question type, and religious faith strength, simple effects of condition (synchronous—asynchronous), conditional effects of SCSRFQ were estimated at representative values of the distribution (Mean—2.5 SD, Mean, and Mean + 2.5 SD). All reported *P*-values are Holm-corrected. For **ownership** and **location**, a robust effect of condition was observed across all levels of religious faith strength scores. For ownership, the condition effect was significant at low religiosity (*Estimate* = 2.10, *SE* = 0.65, *df* = 682, *t* = 3.23, *P* = .015), average religious faith strength (*Estimate* = 2.39, *SE* = 0.24, *t* = 9.92, *P* < .001), and high religious faith strength (*Estimate* = 2.68, *SE* = 0.65, *t* = 4.13, *P* = .001). Similarly, for location, the effect of condition remained significant across the religious faith strength scores spectrum (low: *Estimate* = 2.50, *SE* = 0.65, *t* = 3.86, *P* = .002; average: *Estimate* = 2.20, *SE* = 0.24, *t* = 9.15, *P* < .001; high: *Estimate* = 1.90, *SE* = 0.65, *t* = 2.94, *P* = .038). **No significant modulation by** the religious faith strength was observed for the dimensions of affect, **deafference, or disownership.** Condition effects in these dimensions were non-significant across all levels of religious faith strength (all Holm-corrected *P*s = 1.000). Interestingly, among participants with stronger religious faith, **motor input failed to reinforce a sense of agency.** Indeed, the **agency** dimension revealed a strikingly different interaction pattern. At low religious faith strength, participants showed a strong and significant condition effect favoring the synchronous condition (*Estimate* = 2.66, *SE* = 0.65, *t* = 4.10, *P* = .001). This effect persisted, though reduced, at average religious faith strength (*Estimate* = 1.21, *SE* = 0.24, *t* = 5.03, *P* < .001). Critically, at higher levels of religious faith, the difference between synchronous and asynchronous conditions was reduced (*Estimate* = −0.24, SE = 0.65, *t* = −0.37, *P* = 1.000), reflecting an attenuation of the condition effect. To further examine this pattern, we conducted a linear mixed-effects model with agency scores as the dependent variable, condition (synchronous, asynchronous) and religious faith strength (SCSRFQ scores) as predictors, and a random intercept for participants’ ID. The interaction between condition and religiosity was significant (*F*(1,62) = 7.11, *P* = .010), indicating that the effect of condition on agency ratings varied as a function of religious faith strength. Simple effects analyses showed that religiosity significantly predicted agency ratings in the asynchronous condition (*Esti*mate = 0.094, *SE* = 0.025, *t* = 3.85, *P* < .001; *M*_−2.5SD_ = −2.31, *M*_mean_ = −0.19, *M*_+2.5SD_ = 1.92), but not in the synchronous condition (*Estimate* = 0.029, *SE* = 0.025, *t* = 1.20, *P* = .232; (*M*_−2.5SD_ = 0.36, *M*_mean_ = 1.02, *M*_+2.5SD_ = 1.68).), suggesting that the interaction was primarily driven by changes in the asynchronous condition. In other words, at higher levels of religious faith, participants reported an illusion of agency even in the asynchronous condition.

*Proprioceptive drift.* Proprioceptive drift was significantly higher in the synchronous compared to the asynchronous condition, *t*(62) = 5.22, *P* < .001, with an estimated mean drift of 43.23 (SE = 4.47) for synchronous and 12.58 (SE = 4.47) for asynchronous condition. No significant main effect of the strength of religious faith strength (*P* = .515) or interaction between condition and the strength of religious faith (*P* = .748) was found.

*Interoceptive sensibility* (BPQ-22)*.* Results showed a significant negative correlation between interoceptive sensibility and the strength of religious faith (*r*_(60)_ = −0.36, *P* = .004). In other words, people with stronger religious faith showed lower interoceptive sensibility. To further explore whether the interoceptive sensibility scores mediated the relationship between the strength of religious faith and the agency component, a mediation analysis was conducted *via* a generalized linear model using the SCSRFQ, BPQ-22, and the difference between the synchronous and asynchronous conditions in agency to better capture the above-described finding. Results confirmed that the strength in religious faith was a significant negative predictor of interoceptive sensibility (*β* = −0.36, *P* = .002), whereas interoceptive sensibility did not significantly predict the agency effect (*β* = 0.05, *P* = .710). The indirect effect of religious faith strength on the agency effect *via* interoceptive sensibility scores was not significant (*β* = −0.02, *P* = .712). In contrast, the direct effect of religious faith strength on the agency effect was significant and negative (*β* = −0.30, *P* = .019). Accordingly, the total effect of religious faith strength on the agency component was also significant (*β* = −0.32, *P* = .008).

## Discussion

The present study examined how the subjective strength of religious faith modulates the mechanisms underlying bodily self-awareness using a multisensory illusion paradigm. We also tested the self-report capacity of perceiving internal bodily signals, that is, interoception sensibility (Garfinkel et al. 2015). Two competing hypotheses were tested: the *attenuation hypothesis*, predicting that religiosity may reduce subjective salience of internal bodily signals and enhance susceptibility to multisensory illusions, and the *stabilization hypothesis*, proposing that religiosity may strengthen higher-order bodily representations, thereby reducing susceptibility to multisensory illusions.

Our findings seem to support the *attenuation hypothesis*: Participants with higher subjective strength of religious faith showed agency ratings that were less sensitive to sensorimotor incongruence, such that asynchronous stimulation no longer reduced perceived agency relative to synchronous stimulation. Moreover, subjective strength of religious faith was inversely correlated with interoceptive sensibility, indicating that more religious individuals were less attuned to internal bodily signals. This pattern suggests that reduced sensibility to interoceptive cues may enhance reliance on visual information to sustain a coherent sense of self.

From a neurocognitive perspective, individuals with high levels of subjective strength of religious faith may rely more heavily on externally guided frameworks for interpreting bodily experience, which are shaped by doctrinal rules, ritual discipline, and long-term bodily regulation (Furey 2012, Hall and Boyatzis 2016, Brandt 2019). These influences may promote a perceptual style in which coherence and control are preferentially anchored to externally available information, particularly visual input, rather than from immediate internal information. The pattern observed in this population may therefore reflect a shift in the relative contribution of sensory signals, whereby exteroceptive evidence—what is seen—is prioritized over the motor feedback—i.e. what is executed and felt. In multisensory contexts such as the Mirror Box Illusion, this weighting may reduce the impact of sensorimotor discrepancies, such that visual input remains the primary driver of the sense of agency even when movements are incongruent. As a result, both synchronous and asynchronous stimulation equally induced the illusion of agency over the reflected hand at high levels of strength of religious faith. These findings are not attributable to a proprioceptive bias, as this measure was congruently modulated by the experimental conditions. In other words, regardless of religious faith levels, participants exhibited a proprioceptive bias in the synchronous but not the asynchronous condition. The dissociation between proprioceptive drift and vividness of illusion of ownership or agency aligns with previous evidence indicating that these measures can be partially independent in Mirror Box Illusion paradigm applications (Crivelli et al. 2023, Salvato et al. 2025a).

Our results showed a negative association between subjective strength of religious faith and interoceptive sensibility. At first glance, this may appear to diverge from previous work reporting a positive association between religiosity and interoceptive sensibility (Van Cappellen and LePage Drummond 2024). However, an important difference between studies concerns how religiosity was conceptualized and measured. Specifically, the previous study assessed religiosity as a multidimensional construct, including not only the centrality of religious belief but also specific body-related beliefs (e.g. viewing the body as sacred), which were shown to be positively associated with interoceptive sensibility. In contrast, the present study focused on the subjective strength of religious faith as a more general and domain-independent construct, without directly capturing such specific beliefs about the body. This difference in operationalization suggests that the two studies may be tapping into partially distinct aspects of religiosity. In particular, associations observed in the previous study may be driven by specific belief systems concerning the body, whereas the negative association observed here may reflect a more general relationship between religious faith and sensibility to internal bodily signals. In addition, methodological differences in assessing interoception may further contribute to variability in findings. The previous study employed the MAIA (Van Cappellen and LePage Drummond 2024), which captures higher-order aspects of interoception, including attention to bodily sensations, their interpretation, and their use in emotional and self-regulatory processes. In contrast, the BPQ-22 used in the present study is more specifically oriented toward perceived sensibility to physiological bodily signals, particularly autonomic and visceral processes. Recent evidence confirms that different self-report measures are not interchangeable: they are only partly correlated and capture different facets of interoceptive sensibility rather than a single unified construct (Desmedt et al. 2022, Vig et al. 2022).

Importantly, the negative correlation between subjective strength of religious faith and interoceptive awareness did not mediate the relationship between subjective strength of religious faith and agency. This suggests that reduced interoception alone does not fully account for the persistence of agency illusions; rather, we speculate that the subjective strength of religious faith may modulate the relative contribution of sensory signals, likely influencing how visual, proprioceptive, and motor information are integrated into a unified sense of control. In this view, the subjective strength of religious faith may reshape the hierarchy of sensory evidence on which agency judgments depend. Within the experimental paradigm, it appears that the motor component is directly modulated by the strength of religious faith, as action incongruence does not reduce the sense of agency for the reflected hand (in the mirror). However, these interpretations should be considered with caution, as we did not directly test the weighting or reorganization of sensory inputs.

## Conclusions

Taken together, these findings suggest that the strength of religious faith may extend beyond belief systems to influence the sensory interplays that contribute to the experience of agency. By relatively privileging external visual information over motor feedback, individuals with stronger religious faith may exhibit a more visually anchored form of self-awareness that appears to remain coherent even in the face of sensory–motor conflict, pointing to the possibility that enduring cultural and spiritual orientations are associated with biologically measurable differences in the architecture of bodily self-awareness.

### Limitations

We conclude by highlighting the limitations and future directions of this work. One limitation is that our study included predominantly Roman Catholic participants. Moreover, the design does not allow causal inferences, and the observed association may reflect bidirectional relationships, whereby differences in bodily experience or interoceptive processing could also influence the development or maintenance of religious belief. Future research could compare religious groups whose practices differentially rely on bodily discipline to test whether specific practices selectively shape interoception, body ownership, or agency. Such rituals, ranging from verbal (e.g. religion-related linguistic habits) to nonverbal (e.g. hiding the body, maintaining fixed postures, and performing repetitive gestures), as well as fasting or prolonged stillness, may shape how people perceive and process sensory-motor bodily signals. A better understanding of these links could illuminate how the experience of faith becomes embodied in the sensory and perceptual mechanisms that ground the sense of self.

Moreover, the present findings may reflect unmeasured variables that modulate the plasticity of bodily self-awareness and interoceptive processing. Individual differences arising from multiple factors that influence the malleability of body ownership or access to internal bodily signals—such as personality traits (Marotta et al. 2016) or political ideology (Ruisch et al. 2022)—could therefore account for part of the observed variance. Because these variables were not assessed in the present study, their potential contribution cannot be ruled out. Future research should therefore examine a broader range of individual-difference variables not considered here, in order to more precisely isolate the specific contribution of strength of religious faith and to better characterize its potential signature within bodily self-awareness.

## Supplementary Material

Appendix_niag041

## Data Availability

The data underlying this article will be shared on request to the corresponding author.
