# Supplementary material for: Strength of religious faith is associated with altered sense of agency during multisensory conflict
Source: Neurosci Conscious. 2026 Jul 30;2026(1):niag041. doi: 10.1093/nc/niag041 (PMC13422634; doi:10.1093/nc/niag041)
Supplement: Appendix_niag041 [file appendix_niag041.docx]

**Appendix**

Subjective experiences of embodiment questions (from Medina et al. (2015)):

1. It felt as though my index fingers were making contact with each other. (Location)

2. It seemed like I couldn’t really tell where my right hand was. (Deafference)

3. I had the sensation that my right hand was numb. (Deafference)

4. I found this experience interesting. (Affect)

5. I found this experience enjoyable. (Affect)

6. It felt like I was in control of [the hand in the mirror/my left hand] (Agency)

7. It seemed like my left hand [was in the same location as the hand in the mirror]. (Location)

8. It felt as though the hand in the mirror was my left hand. (Ownership)
